# Micronutrients Improve Growth and Development of HLB-Affected Citrus Trees in Florida

**DOI:** 10.3390/plants12010073

**Published:** 2022-12-23

**Authors:** Samuel Kwakye, Davie M. Kadyampakeni

**Affiliations:** Citrus Research and Education Center, University of Florida, 700 Experiment Station Rd., Lake Alfred, FL 33850, USA

**Keywords:** *Citrus sinensis*, enhanced nutritional program (ENP), huanglongbing (HLB), micronutrient

## Abstract

Enhanced nutritional programs (ENPs) have improved citrus trees’ growth and development in the era of Huanglongbing (HLB). Studies conducted with variable rates of manganese (Mn) and Iron (Fe) on young HLB-affected citrus trees showed that applying double the standard recommendation increased growth and biomass accumulation. Since HLB is believed to cause deficiency symptoms of micronutrients in citrus trees, it is critical to ensure their optimal levels in the leaves. This could be achieved by soil application of either a Mn rate of 8.9 to 11.5 kg ha^−1^ as MnSO_4_ (31%) for young HLB-affected ‘Valencia’ (*Citrus sinensis* (L.) Osbeck) citrus trees or an Fe rate of 9.6 to 11.8 kg ha^−1^ as Ferrous sulfate heptahydrate (20%) for ‘Bingo’ (*Citrus reticulata*, Blanco) citrus trees. Maintaining optimal levels of these micronutrients may enable citrus trees to carry out photosynthetic activities to ensure growth and development. It may also help the tree in the regulation of various physiological processes as part of the antioxidant enzyme Mn-superoxidase dismutase (SOD). Micronutrient manipulation through variable rates of fertilizer application to influence nutrient availability is an important mitigating factor for HLB-affected citrus trees and an integral component of citrus production in Florida.

## 1. Introduction

During the last two decades, the total citrus production in the United States (US) has declined significantly [1,2,3]. Florida, the second largest citrus producer in the nation, has recorded the greatest reduction of more than 80%, from 13.5 million tons in 1998 to about 2.6 million tons in 2021 [4]. Despite this reduction, citrus remains one of the leading tree crops produced in Florida, contributing about $1.3 billion annually to the state’s economy [4]. Citrus production decline has been largely ascribed to huanglongbing (HLB) or citrus greening disease that was first reported around 2005 [5,6]. Other challenges, such as hurricane incidence, disease and pest infestation, prolonged water scarcity, and market competition, have also contributed to the decline in production [6,7,8,9,10,11]. However, HLB has been the major cause of yield reduction, poor juice quality, and smaller fruit sizes in recent years [1,12].

HLB, which means “yellow shoot disease” in Chinese, is believed to be caused by multiple groups of phloem-limited bacterium that belong to the genus, *Candidatus Liberibacter asiaticus* (CLas) [6,13,14]. HLB is phloem-limited because the bacteria propagate in the Phloem of the tree, where the translocation of minerals takes place [14]. HLB is spread from tree to tree by an insect vector called Diaphorina citri, Kuwayama (Asian citrus psyllid, ACP) [5,6,14]. The ACP completes its life cycle, which consists of eggs, nymphs, and the adult stage, on new growth or on shoot tips [6,14]. Its mode of transmission is by feeding and injecting the bacteria into the phloem of the tree [5,14]. HLB was first found in China in the 19th century and has spread to most parts of the world, thus threatening the global citrus industry [15,16]. After the disease was reported in 2005, HLB was detected in parts of the US, such as Georgia, Louisiana, South Carolina, Texas, and California [6,16,17].

The disease interrupts the physiological functions of citrus trees, including but not limited to translocation of mineral nutrients from one part of the plant to the other, yellow shoot, blotchy-mottled leaves, and branch dieback that affects metabolism and growth of the tree [15,18]. When a citrus tree is HLB infected, there is a decline in roots and fibrous root density, leading to a reduction in nutrient and water uptake, which in turn leads to nutrient deficiency symptoms and a decrease in yield [7,8,19]. Citrus trees affected with HLB usually show deficiencies in manganese (Mn), zinc (Zn), phosphorus (P), calcium (Ca), magnesium (Mg), iron (Fe), and boron (B), and also require a minimum amount of water [2,19,20,21]. Therefore, application rates of micronutrients in general need to be readjusted to ensure their optimal levels in plants to enable growth and development. In addition, considering that the citrus nutrition management guidelines by the University of Florida Institute of Food and Agricultural Sciences (UF/IFAS) were developed prior to HLB in Florida, it is worthwhile to evaluate the rates of application for micronutrients to determine optimal thresholds in a balanced fashion that are therapeutic to tree health [2,20,22].

Currently, HLB has no cure, however, the management programs adapted for HLB include intensive chemical control of the ACP, aggressive removal of HLB-affected trees, enhanced nutritional programs (ENPs), and planting disease-free nursery rootstocks [23]. The frustration of managing citrus trees affected by HLB along with adverse weather conditions is influencing the decisions of some producers to either change crop type or use the land for a nonagricultural activity, contributing to the rapid decline of citrus production in Florida. The purpose of this review was to give an overview of how HLB has affected citrus nutrition, specifically, micronutrient dynamics in citrus trees, and assess research on the use of micronutrients as therapies for HLB-affected trees.

By far, ENPs appear to mitigate and help manage citrus trees in the era of HLB [2,18,20,22,24]. The nutritional level of plants and their defense mechanism can be highly interrelated, as some studies have shown the benefit of micronutrients on both the health and natural defense of citrus crops, in response to the action of diverse types of pathogens [2,18,20,25]. As mentioned earlier, there are many factors that could limit citrus fruit yields including diseases and hurricanes [3,7,8,10,19]. However, inadequate nutrition, especially during the critical growth period of citrus trees may not only reduce yield but also produce fruits with poor juice and size quality [1]. According to Morgan et al. (2016), the interaction between HLB-affected trees and nutrient uptake can vary, resulting in different nutrient concentrations in plant tissues, depending on the mobility of that nutrient [3,18].

For the moment, mitigating HLB and keeping citrus trees healthy will require more than one approach until a cure is found [9,19]. Some citrus growers are now customizing fertilization with pest control practices, to keep trees productive [14,26,27,28,29]. The objective of this review highlights efforts made by researchers to mitigate the negative impacts of HLB and to keep citrus trees productive, with emphasis on micronutrients. Since there has not been any known review about how ENPs could be used as a therapy for HLB in Florida, this review may identify research in the area of citrus nutrition and the gap in knowledge to improve citrus production in the era of HLB.

## 2. Efforts to Mitigate the Effects of HLB

### 2.1. Resistant or Tolerant Rootstock

Rootstocks have played a crucial role in helping to maintain citrus trees’ productivity in the era of HLB [30]. Although there has not been a resistant rootstock to HLB, some rootstocks have been observed to provide more tolerance to the disease relative to others [31]. A study on 15 different rootstocks revealed that Volkamer lemon and US 897 rootstocks have shown some level of tolerance that might enable young trees to withstand the damaging effects of HLB [31]. Since it has been shown that greater than 40% of the citrus root is damaged before HLB symptoms are observed in above-ground tissues [7,32], a rootstock that is tolerant to HLB would make a significant difference as far as the growth and development of the tree is concerned [31]. The capability of some rootstocks to adapt well to different soil conditions makes them more tolerant to HLB than others [30,31]. However, rootstock performance may depend on several factors including the drainage system of the soil, pH, and salinity, among others [30]. Soil pH, specifically, plays an important role in the absorption of water and nutrient and root growth [33]. By far, there is no rootstock that is resistant to HLB, however, there is a possibility that rootstocks show some form of tolerance to HLB if they are able to withstand the adverse conditions stated above [31].

### 2.2. Use of Protective Covers

Another tool that has been key to providing young citrus trees (1–4 years old) a lifeline against infestation by ACP is the individual protective cover (IPC) [12]. These are screens that fit over each tree to avoid feeding by the ACP as long as the prevention method lasts [34]. The IPC is said to be one of the effective strategies to prevent the incidence of ACP, which in turn keep citrus trees free from HLB [12]. The IPC is made of either high-density polyethylene or polyvinyl that has a mesh size smaller than the ACP [12,30]. The use of IPC is only partially adopted by commercial citrus growers because the method could be expensive and might increase production costs [30]. Therefore, it is economically applicable for fresh fruit producers who typically have relatively high returns [12,30]. The IPC has successfully excluded ACP from contact with the leaves [12].

### 2.3. Chemical Control of ACP

Intensive chemical control programs against ACP have been deemed necessary to combat HLB by growers in Brazil and Florida [35]. The use of insecticides is traditionally among the early and widely adapted strategies used to control the population of insect infestation in crop production to minimize the spread of plant disease [30,35]. Applying insecticides at critical flushing periods can significantly reduce populations of ACP, therefore, routine applications of insecticides in Florida citrus have been recommended to control ACP [13]. For insecticide application to control ACP, timing is crucial because the active chemical may either work on the adult, the nymph, or both [13,30]. Fenpropathrin for instance kills ACP adults within minutes, even before they can acquire and transmit the disease [30,35]. Therefore, it is critical to know which stage to apply for effective results [13]. For the control of ACP, insecticides such as imidacloprid, Cholorpyrifors 4E, fenpropathrin, Dimethoate 400, Endosulfan, and Malathion, among others, have been labeled for use in citrus [13,30,35]. However, frequent use of one class of insecticides and application of higher doses have caused the ACP to develop strategies that detoxify the active ingredient, making the chemical harmless to the ACP [30]. This has rendered most insecticides to be noneffective for the control of ACP [30,35]. Some investigators have confirmed resistance of ACP to insecticides, for example, in the US, Brazil, and Vietnam [36,37,38]. There have been reports on the use of natural predators such as lady beetles (Coleoptera: Coccinellidae), syrphid flies (Diptera: Syrphidae), lacewings (Neuroptera: Chrysopidae, Hemerobiidae), and spiders (Araneae) [16,23,36]). Although, not much has been reported on the extent to which these predators reduce ACP infestation to be considered a biological control agent [36].

### 2.4. Use of Enhanced Nutritional Program(s)

There has been multiple research that supports the use of ENPs to mitigate the damaging effects of HLB [18,22,24,39,40,41,42]. When citrus trees are infected by HLB disease, they show signs of micronutrient deficiencies (for example zinc, manganese, and iron) and this may be because the severity of HLB causes phloem plugging, thus limiting nutrient translocation within the plant [14,25] and as a result damages more than 40% of the fibrous roots [7,32], which may, in turn, inhibit nutrient absorption. Considering the importance of micronutrients such as Mn and Fe in photosynthesis, and as a component of Mn superoxide dismutase (Mn-SOD); in defense of the plant against stress, a deficiency of Mn may be damaging to the total development of the tree [43]. Some researchers in Florida found enhanced foliar micronutrient application to increase yield as compared to the standard micronutrient application, although, in their study, it was not cost-effective [2,40]. Researchers indicated that Mn with sulfate increased yield when compared to other macro and micronutrients applied [18].

## 3. What Is Known

Studies have shown that ENPs are an effective way to mitigate HLB since the affected trees exhibit nutritional imbalances [3,26]. The supply of either Zn, Mn, or Cu through the leaves and soil tends to help HLB-affected trees avoid the negative impact of the disease [44]. Researchers evaluated the effects of Zn, Mn, and Cu on the physiological growth of HLB-affected and healthy control trees [44]. They observed that the citrus trees infected with CLas generally had lower dry-weight biomass irrespective of the subjected treatment when compared with the healthy trees [44]. These results agreed with that of other researchers in Florida where they observed a significantly reduced dry-weight biomass for 2 year-old HLB-affected sweet orange trees that were subjected to variable Mn rates application [22]. In 2016 and 2017, a study conducted to evaluate the interaction of HLB and foliar application of Cu on the growth and nutrient acquisition of sweet oranges also showed reduced dry-weight biomass [20]. It seems that, once CLas infects the tree, growth is retarded to some extent, and this may be because of an interruption of metabolism, due to the starch accumulation in the phloem [5].

In 2019, researchers conducted a study on the therapeutic effects of Mn and other micronutrients on HLB-affected sweet oranges and observed that applying four (4×) times the standard recommendation rate was therapeutic for the 8–10 year-old HLB-affected trees [42]. For their study, they reported that the citrus trees subjected to 4× the standard recommendation had a high cycle threshold (Ct) relative to the other treatments [42]. However, a study conducted by other researchers on the growth and development of 1–3 year-old HLB-affected sweet orange and mandarin trees subjected to variable rates of Mn and Fe, respectively observed trunk and height increase with rates equivalent to double (2×) the standard recommendation [22,24]. For these same studies, they observed no impact of the micronutrients on Ct values [22,24]. This helps in the understanding that there have been some variabilities in terms of micronutrient rates for better growth and their role with Ct values and the age of trees.

In some cases, foliar Mn application at a rate of 3× the standard application showed a 45% yield increase when compared with the unsprayed control [3,18]. However, researchers observed a 25% yield reduction when trees were subjected to 6× the standard recommendation with an increase in canopy size at the expense of yield [18]. This suggests that modifying the traditional micronutrient recommendations in Florida seems to be appropriate for sweet orange trees affected by HLB [2,3,26]. A follow-up study on the latter observed that the applied micronutrients showed significant variation in seasonal root growth [2]. For example, there was a reduction in fine root length and density following the application of 3× the standard application rates for micronutrients [2]. Moreover, other researchers observed that foliar application of Fe^2+^ can restore the growth of citrus trees affected by greening [45]. In their study, the HLB-affected trees subjected to Fe^2+^ foliar application showed faster growth than the untreated control [45].

**Figure 1:**
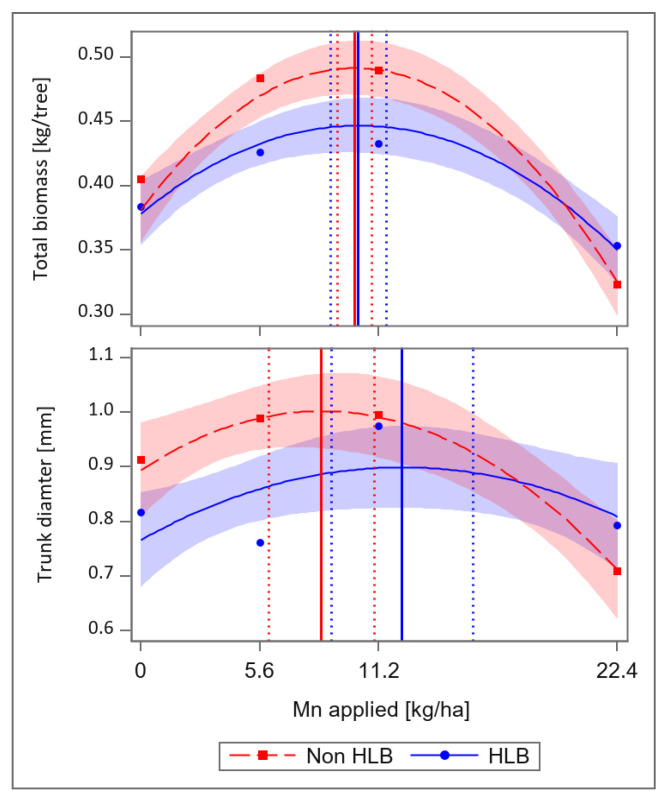
Maximum dry-weight biomass (kg/tree) and trunk diameter (mm) in response to Mn rates for Huanglongbing (HLB)-affected 2 year-old ‘Valencia’ [*Citrus sinensis* (L.) Osbeck] trees. Vertical solid line represents the Mn rate at which maximal response was achieved, and vertical dotted lines represent the lower and upper 95% confidence interval. Rates were 0.0 (Control), 5.6 [Standard recommendation-1× by University of Florida Institute for Food and Agricultural Sciences (UF/IFAS)], 11.2 (2× UF/IFAS rate), and 22.4 kg ha^−1^ Mn (4× UF/IFAS rate). Total sample (N = 12) for each category.

**Figure 2:**
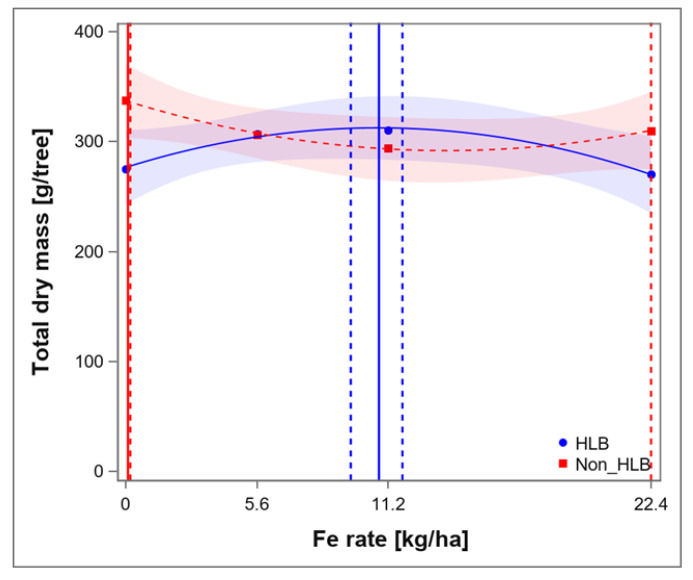
Maximum dry-weight biomass (g/tree) in response to iron (Fe) rates for huanglongbing (HLB)- affected 2 year-old ‘Bingo’ (Citrus reticulata, Blanco) trees. Vertical solid line represents the Fe rate at which the maximal response was achieved. Vertical dotted lines represent the lower and upper 95% confidence interval (CI). Rates were 0.0 (control), 5.6 (standard recommendation, 1×, by University of Florida/Institute of Food and Agricultural Sciences), 11.2 (2×), and 22.4 (4×) kg ha^−1^ Fe. Total sample (N = 12) for each category.

From the information gathered, it appears that the HLB-affected trees require more micronutrients than what is traditionally recommended for citrus production in Florida to achieve optimum nutrition. It has also been observed that foliar application of micronutrients to supplement soil-applied nutrients is able to correct deficiencies for HLB-affected trees [22,24,39,42]. HLB can impact yield due to poor growth and development, because of reduced root biomass and nutritional deficiencies [6,7,8,19], it is important that growers supply optimum nutrition while the tree is still young (between 1–4 years old). For this reason, knowledge about how much micronutrient is required of the young tree may be necessary for growth and biomass accumulation [22,24]. Two recent studies provided the optimal ranges of Mn and Fe at which growth and biomass are at a maximum (Figure 1 and Figure 2) [22,24]. Thus, if citrus growers in Florida apply Mn and Fe at this rate, the tree may have the capability to produce and hold fruits and this might improve the overall yield.

## 4. Enhanced Nutritional Program for Citrus Production

Enhanced nutritional programs (ENPs) are slow- or controlled-release, liquid or dry soluble granular fertilizers that contain all or most essential macronutrients and micronutrients to provide the citrus trees with readily available nutrients throughout the production season to mitigate the debilitating impacts of HLB. There are three major criteria that qualify a mineral element to be considered an essential plant nutrient [46,47]. These include (1) a given plant must be unable to complete its life cycle in the absence of the mineral element, (2) the function of the element must not be replaceable by another mineral element, and (3) the element must be directly involved in plant metabolism, for example, as a cofactor of an enzyme [46]. This means that all essential mineral elements for a citrus tree are deemed important, and if one of them is deficient, it can limit the growth potential of the tree. Similar to that of many other higher plants, citrus trees require all essential nutrients in their right proportion [1].

The goals of optimal nutrient management are to (1) ensure that plants have optimal levels of essential nutrients for growth and development throughout all critical growth stages, (2) guarantee an adequate supply of all essential nutrients either through plant roots or leaves, and (3) ensure that soil physical and chemical properties favor nutrient absorption by plant roots [1,46]. It is well understood that a growing plant may have already lost its potential while deficiency symptoms are observed on the leaves. Therefore, it is the goal of any nutrient management program to test plant leaf tissue to ensure that the levels of all essential nutrients are optimized.

## 5. Why Micronutrients Matter for HLB-Affected Trees

Manganese is an essential element for plants, intervening in several metabolic processes, mainly in photosynthesis and as an enzyme antioxidant cofactor [46,48]. Reduced Mn (Mn^2+^) form is the only available metal form for plants. It is taken up through an active transport system in epidermal root cells and transported as divalent cation Mn^2+^ into the plants [46,49,50]. According to past research, Mn has a profound influence on three physiological (metabolic) functions: (i) photosynthesis, particularly electron transport in photosystem II and chloroplast structure, (ii) N metabolism, especially the sequential reduction of nitrate, and (iii) aromatic ring compounds as precursors for aromatic amino acids, hormones (auxins), phenols, and lignin [50,51]. The concentration of Mn in the soil may be controlled by chemical complexes formed by Mn^2+^ due to low or high pH [52]. At higher soil pH (up to about pH 8), autooxidation of Mn^2+^ is over MnO_2_, Mn_2_O_3_, and Mn_3_O_4_, which are not normally available to plants [49,52,53]. Manganese is an important oligo element involved in the regulation of many different physiological processes as well as part of the antioxidant enzyme Mn-SOD [54]. Manganese deficiency greatly affects photosynthesis; however, visual symptoms occur when plant growth is severely depressed [55]. Deficiency symptoms are observed in newly emerged leaves because of low phloem mobility of Mn that prevents remobilization of Mn from older to younger leaves [55]. In addition, Mn deficiency causes reductions in lignin concentrations in plant roots [52,55]. Research has revealed that Mn deficiency in citrus may significantly reduce yield and fruit color, and the fruit may become smaller and softer than normal [1].

Iron (Fe) is a transitional element that is characterized by the relative ease by which it may change its oxidation state and by its ability to form complexes with different ligands [46]. This variability expressed by Fe is essential in biological redox systems [46]. Iron as a micronutrient is required by most plants in small quantities. It is well known for its metabolic processes such as deoxyribonucleic acid (DNA) synthesis, photosynthesis, and respiration [56]. It is also a constituent of many electron carriers and enzymes, and therefore, important in plant metabolism [56]. The presence of Fe in iron-containing heme proteins makes its levels in the plant critical in the electron transfer chain e.g., cytochromes [57]. Cytochromes are found in the electron transfer systems in chloroplasts and mitochondria [46,57]. Other heme enzymes are catalase and peroxidases [46]. It is reported that, under conditions of Fe deficiency, the activity of both types of enzymes declines [46].

Although Fe is abundant in the soil, it is mostly in a complex form, and plants absorb Fe by an active process, thus, by giving out energy to reduce Fe^3+^ to Fe^2+^ to make it available for absorption in the rhizosphere [57,58]. Plant iron absorption is also dependent on soil pH and redox potential [58]. At lower pH, Fe is readily available to plants, however, in aerobic soil conditions and high pH soils, Fe is in the form of insoluble ferric oxides [46,58]. Since HLB weakens the tree’s immune system [1,3] and contributes to the loss of more than 40% of the fibrous root system [32], it is a concern that affected trees may not exert enough energy to absorb required Fe, hence, affecting the rate of Fe absorption [7,32,46,58]. It is therefore critical to provide an adequate amount of Fe in the form that is readily available in the rhizosphere to increase their chances of being absorbed [1,24].

Iron deficiency is characterized by chlorosis in young leaves, which is not only associated with the decline of chlorophyll and ßcarotene, but also with changes in the expression and assembly of other components of the photosynthetic apparatus [46,58]. Due to the low solubility of the oxidized ferric form in an aerobic environment, Fe in the soil is mostly not available to plants [59]. When the plant is deficient in Fe, the ferredoxin content is decreased to a similar extent as the chlorophyll content, and the fall in ferredoxin level is associated with a lower nitrate reductase activity [46,60].

Low pH and moisture conditions could trigger Fe toxicity and may be a serious problem for the growth and development of citrus [1,46,58]. Even though this condition is predominantly observed in waterlogged soils and in the event of heavy rainfall or excess irrigation [46], other researchers have reported that the iron catalyzed formation of oxygen-free radicals in the chloroplasts can cause Fe toxicity under dryland conditions [61].

## 6. What Could Be Done in the Future?

The USDA-NASS projected that there would be about a 32% decline from the previous year’s total production (2.6 million tons, 2021–2022) for the 2022–2023 citrus production season [4]. This projection was made in September 2022 before hurricane Ian hit Florida. Thus, the actual production could be much lower [62,63]. Pathogens can alter the nutrition of citrus trees in diverse ways that are reflected in the symptoms of the disease. Some pathogens may immobilize nutrients in the rhizosphere or in infected tissues and interfere with translocation and utilization efficiency [64]. However, mineral nutrients confer crop resistance and tolerance to diseases [62]. Nutrient manipulation through fertilization, or modification of the soil environment to influence nutrient availability, may be a useful cultural control for plant disease and an integral component of production agriculture [62,64]. In general, it is expected that citrus demonstrates some symptomology from pathogen attack or disease infection, especially when they are deficient in one or more micronutrients since most micronutrients function to intervene in the activity of chemical processes (redox process, ROS production, etc.) or enzyme biosynthesis [65,66].

This review focused on the impact of essential micronutrients on mitigating HLB to promote citrus tree growth and development, which in turn might improve yield. It is expected that micronutrient fertilization for HLB-affected citrus trees will be updated in view of recent study findings, to ensure that tree productivity is improved. It may be ideal to investigate the impact of variable rate application of other micronutrients such as Zn, Cu, and B on HLB-affected citrus trees. The latter, among other previous information provided in this review, might be a critical addition to citrus nutrient management guidelines. Another way to mitigate the effects of HLB is to launch site-specific research into other methods of fertilizer applications to maximize nutrient use efficiency. This could be either a single application method, such as fertigation only, or a combination of fertigation supplemented with either foliar or granular application to ensure better tree growth and development.

## Data Availability

The maximum dry-weight biomass in response to Mn rate dataset is publicly available from HORTSCIENCE 57(3): 360–366 (https://journals.ashs.org/hortsci/view/journals/hortsci/57/3/article-p360.xml. It was accessed on 10 September 2022). The maximum dry-weight biomass in response to Fe rate dataset are available from HORTSCIENCE 57(9) (https://journals.ashs.org/hortsci/view/journals/hortsci/57/9/article-p1092.xml, accessed on 10 September 2022).
